# The effects of estradiol levels on crossmodal perception: a study on the sound induced flash illusion in healthy and menstrually related migraine individuals

**DOI:** 10.1007/s10072-023-06744-6

**Published:** 2023-03-15

**Authors:** Simona Maccora, Nadia Bolognini, Carlo Mannina, Angelo Torrente, Luisa Agnello, Bruna Lo Sasso, Marcello Ciaccio, Guido Sireci, Filippo Brighina

**Affiliations:** 1 Section of Neurology, Department of Biomedicine, Neuroscience, and Advanced Diagnostic (BiND), University of Palermo, Via del Vespro 124, 90127 Palermo, Italy; 2 Neurology Unit, ARNAS Civico Di Cristina and Benfratelli Hospitals, Palermo, Italy; 3 Department of Psychology, University of Milano-Bicocca, Milan, Italy; 4 Neuropsychological Laboratory, IRCCS Istituto Auxologico Italiano, Milan, Italy; 5 Institute of Clinical Biochemistry, Clinical Molecular Medicine and Clinical Laboratory Medicine, Department of Biomedicine, Neuroscience, and Advanced Diagnostic (BiND), University of Palermo, Palermo, Italy; 6 Central Laboratory of Advanced Diagnosis and Biomedical Research (CLADIBIOR), University of Palermo, Palermo, Italy; 7 Department of Biomedicine, Neuroscience, and Advanced Diagnostic (BiND), University of Palermo, Palermo, Italy

**Keywords:** Sound-induced flash illusions, Menstrually related migraine, Cortical excitability, Estradiol

## Abstract

**Objective:**

The sound-induced flash illusion (SIFI) is a valid paradigm to study multisensorial perception. In the “fission” SIFI, multiple flashes are perceived when observing a single flash paired with two or more beeps. SIFI is largely dependent on visual and acoustic cortex excitability; in migraine, dysfunctional cortical excitability affects SIFI perception. Since estrogen peak occurring during ovulation can increase neuronal excitability, the present study aims to verify whether cortical excitability shifts linked to the menstrual cycle could influence SIFI.

**Methods:**

In a comparative prospective study, we tested the effect of estrogens on crossmodal perception using the SIFI. We recruited 27 females in reproductive age, including 16 healthy and 11 menstrually related migraine females, testing their proneness to SIFI on day 14 (high estradiol) and day 27 (low estradiol) of menstrual cycle.

**Results:**

Women on day 14 reported less flashes than on day 27 (*p* = 0.02) in the fission illusion, suggesting a pro-excitatory effect of estradiol on visual cortex excitability during ovulation. Moreover, we confirmed that migraine women perceived less flashes (*p* = 0.001) than controls, independently from cycle phase. Non-migraineurs women significantly reported more flashes on day 27 than on day 14 (*p* = 0.04).

**Conclusions:**

This study suggests that estradiol may influence the multisensory perception due to changes of visual cortex excitability, with high estradiol peak leading to increased visual cortical sensitivity during ovulation in non-migraineurs. Visual cortex hyperresponsiveness, here reflected by reduced SIFI, is not influenced by estradiol fluctuations in migraine women, as shown by reduced fission effects on day 14 and 27.

## Introduction

Variations of gonadal hormones estradiol and progesterone can affect cognition, mood, emotion, and social behavior throughout the menstrual cycle and contribute to modulate the delicate balance between excitatory and inhibitory processes in the central nervous system. Estradiol is known to amplify excitatory transmission by enhancing *N*-methyl-d-aspartate (NMDA) glutamate receptor activity and suppressing γ-aminobutyric acid (GABA_A_) transmission, while progesterone potentiates inhibition through GABA_A_ receptors [1–4]. Estradiol can also modulate opioid [5] and serotoninergic systems [6].

Smith et al. [7, 8] first investigated the role of sex hormones variations on primary motor cortex excitability by means of paired transcranial magnetic stimulation (TMS). A first study [7] showed that intracortical inhibition is more pronounced during the luteal phase than in the follicular phase of the cycle, suggesting that progesterone could have an inhibitory effect. In order to detect the effects of estradiol, the same authors conducted a second study [8], showing a facilitatory effect of estradiol during the follicular phase of ovarian cycle. Using 5-Hz repetitive transcranial magnetic stimulation (rTMS), other authors showed that motor-evoked potential (MEP) amplitude increased during late follicular phase (day 14: high estradiol, low progesterone) whereas there was not any facilitation on early follicular phase (day 1: low estradiol and progesterone) [9]. Although further studies failed to demonstrate that the menstrual cycle modulates cortical excitability [10, 11], Schloemer et al. [12] proved that intracortical inhibition by paired-pulse TMS is reduced during high estradiol levels in both somatosensory and visual cortices, suggesting an excitatory role of estradiol on intracortical sensory processing.

Crossmodal illusions can be easily used to assess multisensory interaction linked to cortical excitability changes in healthy and pathological conditions [13]. In particular, an indirect measure of cortex excitability in visual and auditory areas can be derived by the study of the sound-induced flash illusion (SIFI), which comprises two illusory effects: the fission illusion, namely the perception of multiple illusory flashes occurring when a visual stimulus (“flash”) is presented with two or more acoustic stimuli (“beeps”); the fusion illusion occurs when two or more flashes are presented with one single flash, leading to a reduction of the number of perceived flashes [14, 15]. The link between such fission and fusion illusions and cortical excitability has been demonstrated by using transcranial direct current stimulation (tDCS): anodal tDCS over the visual primary cortex or cathodal tDCS over the auditory cortices can reduce or increase the SIFI, respectively [16]. Moreover, neuropsychological evidence showed that fission and fusion effects are impaired by occipital stroke with visual field loss but are preserved after parietal cortical damages associated to the syndrome of spatial neglect. The size of occipital cortical lesion predicts the magnitude of the fission effects [17].

On such bases, we have demonstrated that episodic migraine patients, especially those with aura and during the attack, present with a reduced susceptibility to the SIFI, underlying a condition of visual cortex hyperexcitability [18]. This pattern is even more evident in chronic migraine patients [19].

Menstrual migraine affects about 20–25% of female migraine patients with greater disability associated with perimenstrual attacks [20, 21]. Menstrual migraine is a broad term including two clinical conditions: pure menstrual migraine with exclusively perimenstrual attacks occurring on or between 2 days before (− 2) and 3 days (+ 3) after menstruation onset, but no migraine at any other time of the cycle and menstrually related migraine with attacks not strictly limited to the premenstrual period (International Classification of Headache Disorders, 2018) [22].

Estrogen withdrawal and prostaglandin release occurring on the perimenstrual days of cycle are thought to be the two main mechanisms involved in the pathophysiology of menstrual migraine attacks. In the premenstrual phase of menstrual cycle, the decline of estrogen concentrations is associated with migraine; moreover, a single injection of estradiol delays the drop in estrogen and the migraine attack onset [23–25]. During menstruation, prostaglandins released from the endometrium play a role in migraine pathophysiology as suggested by the ability of injection of prostaglandins to induce migraine attacks [26] and the greater risk of migraine in patients suffering from dysmenorrhea in which pain is mediated by prostaglandins [27].

Following these premises, we envisaged testing if hormonal variations related to menstrual cycle had modulatory effects on crossmodal illusory perception as indexed by the SIFI, therefore underlying effects on cortical excitability. For this purpose, we assessed the role of estradiol on day 14 (late follicular phase corresponding to ovulation: high estradiol, low progesterone) and day 27 (late luteal phase: low estradiol and progesterone) in healthy young women. A secondary objective was to evaluate the influence of sex hormone variations on SIFI in menstrual-related migraine.

## Materials and methods

### Participants

This was a prospective monocentric comparative cohort study conducted at the Neurology Unit of the University Hospital Policlinico of Palermo. Subjects were enrolled from October 2013 to June 2022.

Participants, to be enrolled in this study, needed to be female and have regular menstrual cycles, to be healthy or to have menstrually related migraine but without being under prophylactic migraine treatment and not assuming oral contraceptives. Participants were all right-handed, had normal corrected-to-normal vision and normal hearing, and were naïve to the aim of the research. Healthy controls should not have any family history of migraine.

All healthy and migraine individuals were examined by an expert neurologist. Patients with menstrually related migraine included in the study were diagnosed with menstrually related migraine without aura (A1.1.2), according to the criteria of the International Headache Society (2018) [22]. The exclusion criteria for both groups were menstrual irregularities, hormonal treatment within 6 months prior to enrollment to the study, neurological, systemic, or psychiatric disorders.

The final sample comprised 27 participants: the healthy control group comprised 16 females (mean age = 26 years; mean education = 19 years), the migraine group 11 females (mean age = 29 years; mean education = 18 years).

With respect to patients with menstrually related, the following clinical data were recorded: disease duration, monthly attack frequency, attack duration, attack intensity in a numeric rating scale (NRS) from 0 (no pain) to 10 (worst pain).

Duration of migraine disease was 9.7 ± 6.5 years (mean ± SD), monthly attack frequency was of 1.7 ± 0.8, attack duration was of 22.1 ± 18.1 h, and intensity of headache attacks (NRS) was of 7.1 ± 1.0.

Additionally, on day 14 and day 27 of menstrual cycle, the levels of estradiol and of progesterone were obtained from 8 migraine patients and 8 healthy women: determination of estradiol and progesterone levels showed normal hormone profiles consistent with normal ovulatory cycle (day 14, estradiol = 91.4 ± 91.6 pg/ml, progesterone = 4.8 ± 11.9 ng/ml; day 27, estradiol = 33.9 ± 27.7 pg/ml, progesterone = 5.0 ± 6.6 ng/ml).

The ethics committee of University of Palermo approved this study, and all participants gave their written informed consent, according to the ethical standards of the Declaration of Helsinki as revised in 2013 [28].

### SIFI task and experimental procedure

Stimuli and procedure were adapted from the original study by Shams et al. [14, 18, 19, 29]. In a dimly illuminated room, participants sat ~ 57 cm in front of a CRT computer monitor (Samsung SyncMaster 1200NF: resolution 1024 × 768, refresh rate 75 Hz), with their eyes aligned with the center of the screen, and their head supported by a chinrest. Two speakers were located beside the screen, aligned with the flashes. Each trial began with the appearance of a white fixation cross, displayed at the center of a black screen (luminance: 0.02 cd/m^2^). At the eccentricity of 5° of visual field, a white disk subtending 2° was flashed one to four times. The following stimuli were presented: single flash trials (1F), accompanied with 0–4 beeps (B) (i.e., 1F0B, 1F1B, 1F2B, 1F3B, 1F4B), which give rise to the fission illusion; multiple flash trials (from 2 to 4F), accompanied with 0 or 1 beep (2F0B, 3F0B, 4F0B, 2F1B, 3F1B, 4F1B), for the fusion illusion. Hence, the total number of conditions was 11. Each flash (luminance: 118 cd/m^2^) and beep (at 80 dB SPL) had durations equivalent to one screen refresh (13 ms). The first beep was followed by the first flash after 26 ms. The interval (stimulus onset asynchrony, SOA) was 65 ms (5 refreshes) between two flashes, and 52 ms (4 refreshes) between two beeps. The participants’ task was to judge the number of flashes seen on the screen. Each condition was repeated 8 times, for a total of 88 trials, given in a random fixed order. The total duration of the task was about 5 min. At the beginning of each session, 10 practice trials were administered, and not included in the subsequent analysis.

All patients were tested in a migraine attack–free period which was defined as at least 72 h before and after the last migraine attack.

### Statistical analysis

Statistical analysis has been performed using the software Statistica 8.

To assess the fission illusion, we analyzed the number of perceived flashes in 1-flash (1F) trials (combined with 0–4 beeps, 0–4B) using a repeated-measures analysis of variance (rmANOVA), with the between-subjects factor group (controls vs. migraine patients) and two within-subjects factors: cycle phase (day 14 and day 27 of menstrual cycle) and the factor beep (from 1 to 5, i.e., 1F0B, 1F1B, 1F2B, 1F3B, 1F4B). To measure the fusion illusion, the mean number of perceived flashes in every multiple flash trials (from 2 to 4 flashes) was analyzed with a rmANOVA, with the between-subjects factor groups (2 levels) and the within-subjects factors: flash (2, 3, 4 flashes), beep (0 or 1 beep), and cycle phase (day 14 and day 27).

Post hoc comparisons were conducted with the Bonferroni test, and the effect sizes were assessed by calculating the partial eta squared (ρη^2^) to measure the proportion of total variance attributable to a main factor or interaction [30]. All statistical results are displayed in Table 1.

**Table 1:** Fission and fusion sound-induced flash illusions (SIFI) tested by mean perceived flashes. Significant tests (*p* < 0.05) are represented in bold

		F	*p*
Fission illusion	**Beep**	**70.2**	** < 0.0001**
	**Group**	**13.71**	**0.005**
	**Cycle phase**	**5.67**	**0.02**
	**Group × beep**	**2.56**	**0.04**
	**Group × cycle phase**	**4.31**	**0.04**
	**Cycle phase × beep**	**2.89**	**0.02**
	Group × beep × cycle phase	2.33	0.057
Fusion illusion	**Flash**	**60.07**	** < 0.0001**
	**Beep**	**19.72**	**0.0016**
	Group	1.45	0.24
	Cycle phase	2.48	0.13
	**Flash × beep**	**82.09**	** < 0.0001**
	Group × flash	1.16	0.32
	**Group × beep**	**7.40**	**0.011**
	Group × flash × beep	1.22	0.30
	Group × cycle phase	0.006	0.94
	Flash × cycle phase	0.61	0.54
	Flash × group × cycle phase	2.87	0.06
	Beep × cycle phase	0.11	0.74
	Beep × group × cycle phase	1.89	0.18
	Flash × beep × cycle phase	0.87	0.42
	Flash × beep × group × cycle phase	1.045	0.36

## Results

### Fission illusion

**Fig. 1:**
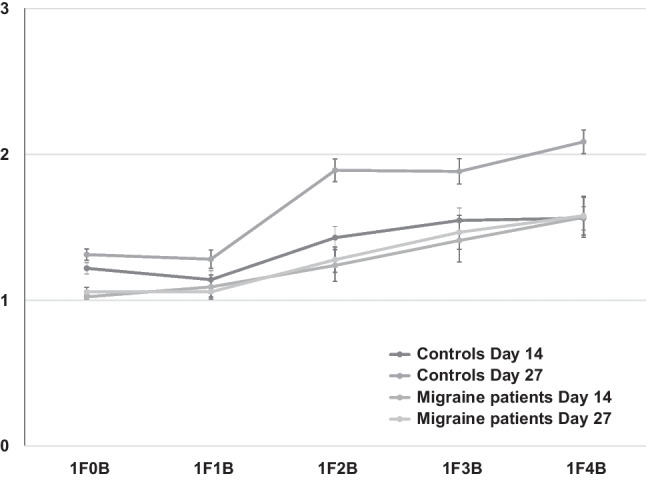
Mean seen flashes and standard errors (bars) in fission trials (1 flash combined to 0–4 beeps) reported by healthy women and menstrually related migraine women on day 14 and 27 of the menstrual cycle

As illustrated by Fig. 1, when a single flash is presented along with multiple beeps, the number of perceived flashes is increased giving rise to the fission illusion. This illusory effect was detected in our experiment, as shown by the beep (F4.2 = 70.2, *p* < 0.0001, ρη^2^ = 0.58): when two or more beeps are presented with a single flash, participants reported a higher number of seen flashes (1F2B = 1.49; 1F3B = 1.6; 1F4B = 1.72), as compared with the 0-beep trial (1F0B = 1.17, *p* < 0.0001) and the 1-beep trial (1F1B = 1.15, *p* < 0.0001).

We also observed a main effect of cycle phase (F1.5 = 5.67, *p* = 0.02, ρη^2^ = 0.10), showing that on day 14 of menstrual cycle (day 14, high estradiol), women reported less flashes than on day 27 (low estrogen). Furthermore, we explored this effect via 5 one-way ANOVAs, one for each stimulus condition with day of cycle as within-subjects factor. For the 1F0B and the 1F1B condition, we found no effect (F1.52 = 1.47, *p* = 0.23, ρη^2^ = 0.02; F1.52 = 1.04, *p* = 0.31, ρη^2^ = 0.02). In contrast, we observed effects for every illusory condition: 1F2B (F1.52 = 7.72, *p* = 0.007, ρη^2^ = 0.13), 1F3B (F1.52 = 4.14, *p* = 0.04, ρη^2^ = 0.07), and 1F4B (F1.52 = 6.75, *p* = 0.01, ρη^2^ = 0.11).

The rmANOVA also showed a main effect of group (F1.5 = 13.71, *p* = 0.005, ρη^2^ = 0.21): migraine women reported fewer flashes than controls in every condition. We also found significant group × beep interaction (F4.2 = 2.56, *p* = 0.04, ρη^2^ = 0.05) and beep × cycle phase interaction (F4.2 = 2.89, *p* = 0.02, ρη^2^ = 0.05). More importantly, we also observed a group × cycle phase interaction (F1.5 = 4.31, *p* = 0.04, ρη^2^ = 0.08), indicating that differently from healthy controls who perceived more flashes on day 27, migraine patients perceived less flashes on both day 14 and day 27. The group × cycle phase × beep interaction (F4.22 = 2.33, *p* = 0.06, ρη^2^ = 0.04) did not attain the significance level.

### Fusion illusion

**Fig. 2:**
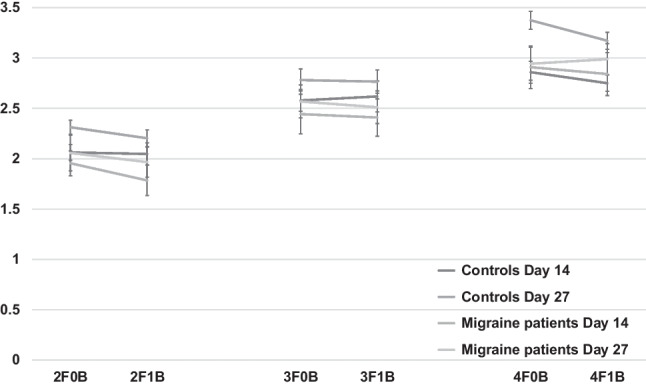
Mean seen flashes and standard errors (bars) in fusion trials (from to 2 to 4 flashes combined to 0–1 beep) reported by healthy women and menstrually related migraine women on day 14 and 27 of the menstrual cycle

On multiple flash trials, participants underreport the number of perceived flashes when a single beep is paired to flashes, the so-called fusion illusion (Fig. 2), as confirmed by the significant effect of beep (F1.25 = 19.72, *p* = 0.0016, ρη^2^ = 0.44). The main effect of flash (F2.5 = 60.07, *p* < 0.0001, ρη^2^ = 0.71) showed an increased number of seen flashes as the number of the presented flashes increased. The interaction flash × beep (F2.54 = 82.09, *p* < 0,0001, ρη^2^ = 0.77) was significant too, but of major relevance is the group by beep interaction (F1.25 = 7.40, *p* = 0.011, ρη^2^ = 0.23): only in healthy controls the presence of a single beep significantly decreases the number of seen flashes while in migraine women group, no difference was found (all ps > 0.9), indicating that migraine patients did not report a reliable fusion illusion.

Group (F1.25 = 1.45, *p* = 0.24, ρη^2^ = 0.05), flash × group (F2.5 = 1.16, *p* = 0.32, ρη^2^ = 0.04), cycle phase (F1.25 = 2.48, *p* = 0.13, ρη^2^ = 0.09), and group × flash × beep (F2.5 = 1.22, *p* = 0.3, ρη^2^ = 0.04) did not reach significance.

## Discussion

This is the first study evaluating the susceptibility to crossmodal illusions, and in particular on the SIFI as an indirect measure of cortical excitability in a group of young women, tested during two different phases of menstrual cycle (ovulatory and premenstrual) in order to determine the effects of different hormonal profiles on crossmodal processing. SIFIs are a valid measure of multisensorial integration and critically depend on cortical excitability of visual and acoustic areas [16, 31, 32].

As for fission effects featuring the SIFI, there was a significant influence of cycle phase on mean perceived flashes under crossmodal conditions. In the late follicular phase (day 14), we found a reduction of SIFI compared to premenstrual phase of cycle (day 27), meaning that estradiol contributes to reduce susceptibility to SIFI in the ovulatory phase likely by increasing occipital cortex excitability. This is in line with previous studies using neurophysiological techniques. Paired-pulse TMS studies [7, 8] have shown that luteal phase is characterized by cortical inhibition as compared to follicular phase and that facilitation is due to estradiol in the late follicular phase. In a repetitive TMS study, Inghilleri et al. [9] demonstrated that corticospinal excitability increases during the late follicular phase (i.e., day 14), indexing a facilitatory effect of estradiol on cortical excitability. Although subsequent studies failed to show any change of intracortical facilitation, intracortical inhibition, and cortical silent period during menstrual cycle [10, 11], the modulating role of sex hormones on intracortical excitability was further demonstrated by paired-pulse suppression [12]: in healthy women, elevated estradiol levels are associated with reduced paired-pulse suppression in both the somatosensory and the visual cortex, suggesting an enhancing effect on sensory areas.

Our data are also in agreement with animal studies [4] showing that estradiol augments glutamatergic transmission and inhibits GABAergic neurons by means of short-term and long-term plasticity mechanisms. As a matter of fact, estrogen can enhance glutamatergic activity in rat Purkinje cells [33], trigeminal ganglion cells activity [34], and NMDA-mediated currents in dorsal root ganglion cells [35]. Moreover, chronic exposure of hippocampal neurons to estradiol can lead to formation of new dendritic spines because of an increased synthesis of brain-derived neurotrophic factor (BDNF) [36]. However, estrogen can have both pronociceptive and antinociceptive effects because it can increase glutamatergic tonus through genomic and non-genomic mechanisms, but also enhances serotoninergic and opioidergic tonus along with a reduction of glutamate reuptake. Actually, estrogen can have direct membrane effects but also modulates gene expression. In trigeminal neurons of female mice, estrogens can modulate pronociceptive neuropeptide expression as shown by increased levels of galanin and neuropeptide Y during the estrus cycle (corresponding to the estradiol peak) [37]. Therefore, we can hypothesize that increased neuronal excitability mediated by estradiol peak is fostered by its genomic effects on the cortex and the nociceptive system; this balance is lost in the premenstrual phase when the estradiol levels fall.

Our results suggest that hormonal profiles influence SIFI perception and that further studies should consider cycle phase in studies on crossmodal processing involving young women in reproductive age.

With respect to migraine, as already shown by previous results [18, 19, 29], we confirmed that independently from the menstrual cycle phase, migraine women perceived less fission effects than healthy women. The fission phenomena of the SIFI depend on visual cortex excitability, along with other association cortices [38–42]. The present finding of reduced proneness to SIFI provides further support on the hypothesis that migraine could be seen as a disorder of multisensorial integration [43, 44] that follows a pathological condition of hyperexcitability of the primary visual cortex [45]. This is in line with previous studies using neurophysiological and neurostimulation techniques. TMS studies showed a magnetic suppression of perceptual accuracy (MSPA) in episodic and chronic migraine patients [46]; the lower MSPA reflects a higher level of cortical excitability due to a disrupted inhibition. In a tDCS study enrolling 16 patients suffering from menstrual migraine, 5 consecutive sessions of cathodal tDCS over the visual cortex 1–5 days prior to menstruation efficiently reduced migraine attacks and increased phosphene threshold [47]. Migraine women in the premenstrual phase also displayed increased amplitude of early components of contingent negative variation as compared to healthy women, suggesting a higher level of cortical excitability before menstruation [48].

Although the present results need to be confirmed in larger samples, we observed that women with menstrual migraine do not show a fluctuation of SIFI during the ovulatory and premenstrual phases of menstrual cycle, as detected in healthy women who, conversely, display a reduction of the fission illusion during the late follicular phase when estradiol peak takes place. We speculate that migraine women present with a baseline hyperexcitability of occipital cortex that cannot be further enhanced by hormonal variation, due to homeostatic plasticity. As a matter of fact, women with menstrual-related migraine present reduced menstrual cyclicity involved in pain perception of trigeminal and non-trigeminal pain stimuli [49]. As postulated by the “neurotransmitter imbalance” theory [50], estrogen can have pronociceptive or antinociceptive effects: when the estradiol levels are high (ovulatory phase), there is a balance between pronociceptive effects mediated by increased glutamatergic tonus, hyperexcitability of trigeminal afferents, and increased synthesis of BDNF and nerve growth factor (NGF) and antinociceptive modulation, due to increased opioidergic and serotoninergic tonus. Instead, during the premenstrual phase of the cycle, when the estrogen levels fall down, the abovementioned pronociceptive mechanisms can prevail, sensitizing neurons to triggers and therefore promoting migraine attacks [51].

While in healthy controls the major susceptibility to fission effects may reflect a reduced cortical excitability in a premenstrual time of the cycle (low estradiol levels), our female migraine sample, showing enhanced cortical excitability, displayed no susceptibility to hormonal fluctuation. This could mean that a stable condition of cortical hyperexcitability is present in migraine and represents a specific pathophysiological tract of the disease, not sensitive to hormonal influences. Alternatively, it could be also hypothesized that further increase of cortical excitability, as that occurring with estrogen peak in normal female, is instead limited in migraine due to activation of homeostatic inhibitory protective mechanisms that take place to avoid detrimental cortical overactivation. Noteworthy, in female rats, cortical spreading depression (CSD) susceptibility is enhanced by high estrogen concentrations while estrogen withdrawal decreased it [52].

This study features itself as exploratory research investigating the link between menstrual cycle and crossmodal perception. The main limit is the small sample size. Moreover, we did not explore the role of progesterone on the perception of SIFI; the investigation of the role of progesterone in the luteal phase could be of relevance to test the potential inhibitory effect of progesterone on SIFI. Moreover, future studies are needed to address the presence of differences between pure menstrual migraine and menstrual-related migraine, enrolling both types of patients.

## Data Availability

The datasets generated for this study are available on request to the corresponding author.

## Abbreviations

ANOVA: Analysis of variance
BDNF: Brain derived neurotrophic factor
CSD: Cortical spreading depression
GABA: γ-Aminobutyric acid
GABA_A_: GABA receptor A
MEP: Motor-evoked potential
MSPA: Magnetic suppression of perceptual accuracy
NRS: Numeric rating scale
NGF: Nerve growth factor
NMDA: *N*-Methyl-d-aspartate
rmANOVA: Repeated measures analysis of variance
rTMS: Repetitive transcranial magnetic stimulation
SIFI: Sound-induced flash illusion
SOA: Stimulus onset asynchrony
tDCS: Transcranial direct current stimulation
TMS: Transcranial magnetic stimulation
